# Meniscal attachment reconstruction combined with high tibial osteotomy in a patient with genu varum and posterior root injury of the lateral meniscus: a case report and brief review of the literature

**DOI:** 10.3389/fsurg.2025.1719884

**Published:** 2025-12-29

**Authors:** Junhu Hou, Xuepeng Ma, Jie Gao, Zhaoyang Zeng, Xingwen Xie, Ning Li

**Affiliations:** 1 Gansu University of Chinese Medicine, Lanzhou, China; 2 Department of Orthopaedics, The Affiliated Hospital of Gansu University of Chinese Medicine, Lanzhou, China; 3 Department of Orthopaedics, Hospital of the 83rd Group Army, Xinxiang, China

**Keywords:** high tibial osteotomy, genu varum, lateral meniscus injury, transtibial tunnel pull-out technique, knee preservation treatment, case report

## Abstract

Rationale: High tibial osteotomy (HTO) is primarily used to treat unicompartmental osteoarthritis. Despite its efficacy, there are strict indications, such as the need for the integrity of the lateral structures, especially the meniscus and cartilage. Here, we report a rare case of a posterior root tear of the lateral meniscus combined with genu varum, demonstrating how reconstructing the posterior root can expand the indications for HTO surgery. Patient concerns: A 58-year-old man complained of “pain in both knees for 5 years, aggravated in the right knee for 1 month.” Diagnoses: A preoperative diagnosis of lateral meniscus injury with genu varum was reached. Interventions: Under normal circumstances, this patient would not have been suitable for HTO. However, we reconstructed the lateral meniscus attachment point using the pull-out technique and then performed HTO. Outcomes: Postoperatively, the weightbearing line of the patient's lower limbs was successfully corrected. Lessons: Aggressive repair of damaged lateral compartment structures combined with HTO can expand the population suitable for knee preservation. Key points: This report describes the first case of posterior root injury to the lateral meniscus combined with knee varus deformity. The patient first underwent lateral meniscus root reconstruction using the pull-out technique, followed by a standard HTO surgery, resulting in satisfactory outcomes.

## 1 Introduction

High tibial osteotomy (HTO) became widely known in the 1970s because of the work of Coventry (1) and Maquet (2). However, at the time, closing wedge tibial valgus osteotomy was performed, which required concurrent fibular osteotomy, and had a high risk of common peroneal nerve injury (3). On this basis, the advantages of tibial opening wedge osteotomy (i.e., HTO) were gradually recognized. In particular, development of the AO Foundation contributed to widespread use of the TomoFix plate (4), an osteotomy fixation system designed based on its internal fixation concept, which caused HTO to gradually gain traction as a mainstream technique (5, 6). This procedure is simple, offers precise orthopedic correction, and has no risk of peroneal nerve injury, thus enabling the effective treatment of medial unicompartmental osteoarthritis of the knee. As more HTO procedures were performed, the indications and contraindications became clearer. Currently, it is believed that HTO is mainly indicated for active patients with unicompartmental osteoarthritis of the knee with genu varum who are aged under 65 years (7–9). Contraindications include severe obesity, lateral meniscus deficiency, degenerative lesions of the lateral compartment (10), and significant limitation of knee motion (especially extension limitation >20°). The desired therapeutic efficacy can only be obtained by strictly grasping the indications and contraindications. However, some rare cases, such as those lying between the indications and contraindications, may cause confusion among clinicians when it comes to choosing a treatment plan. This report describes the first case of an intact lateral compartment cartilage but with a tear in the posterior root of the lateral meniscus due to trauma, in whom transtibial tunnel meniscus attachment reconstruction was innovatively performed, followed by conventional HTO. The operation proceeded smoothly, ideal orthopedic effects were achieved, and the patient was satisfied with the outcome.

## 2 Case presentation

### 2.1 Patient information

A 58-year-old man was admitted to hospital on 28 January 2023 reporting “pain in both knees for 5 years, aggravated in the right knee for 1 month.” The patient experienced pain in both knees with limited mobility for 5 years and self-medicated with oral painkillers, with fluctuating symptoms. He sprained his right knee 1 month ago and the pain worsened. Swelling was reduced by applying medicated plasters and immobilization, but the pain was still evident when walking. The patient was admitted to the orthopedic department of our hospital on 28 January and was diagnosed with “(1) osteoarthritis of both knees; (2) meniscus injury of the right knee.” He was previously fit and had no other history of infectious disease, medical illness, or surgery. The patient had no significant past medical history or family history.

### 2.2 Diagnostic assessment

Upon physical examination: Examinations of the heart, lungs, and abdomen revealed no specific findings. Upon special examination, the right knee showed genu varum and swelling, with a slightly elevated skin temperature. There was obvious tenderness in the medial and lateral joint spaces of the right knee. McMurray's sign was (+), right knee flexion and extension were normal, and no clear abnormalities in the muscle strength, tendon reflexes, or skin sensation of both lower limbs were observed. The pain visual analog scale (VAS) score was 5 points. We also conducted KOOS scores (S = 39.29, *p* = 38.89, ADL = 51.47, Sport = 25, QOL = 25).

The patient's routine blood, urine and stool tests, biochemical tests, coagulation tests, infectious disease tests, and rheumatology tests were all normal. The results of individual tests for abnormalities are shown in Table 1.

**Table 1:** Abnormal test results.

Items	Preoperative	Postoperative
White blood cell count (×10^9^/L)	2.92	4–10
Neutrophil absolute count (×10^9^/L)	1.46	2–7
Urea (mmol/L)	8.7	1.43–7.14
Alkaline phosphatase (U/L)	29	50–136
Adenosine deaminase (U/L)	25.93	4–24

**Figure 1:**
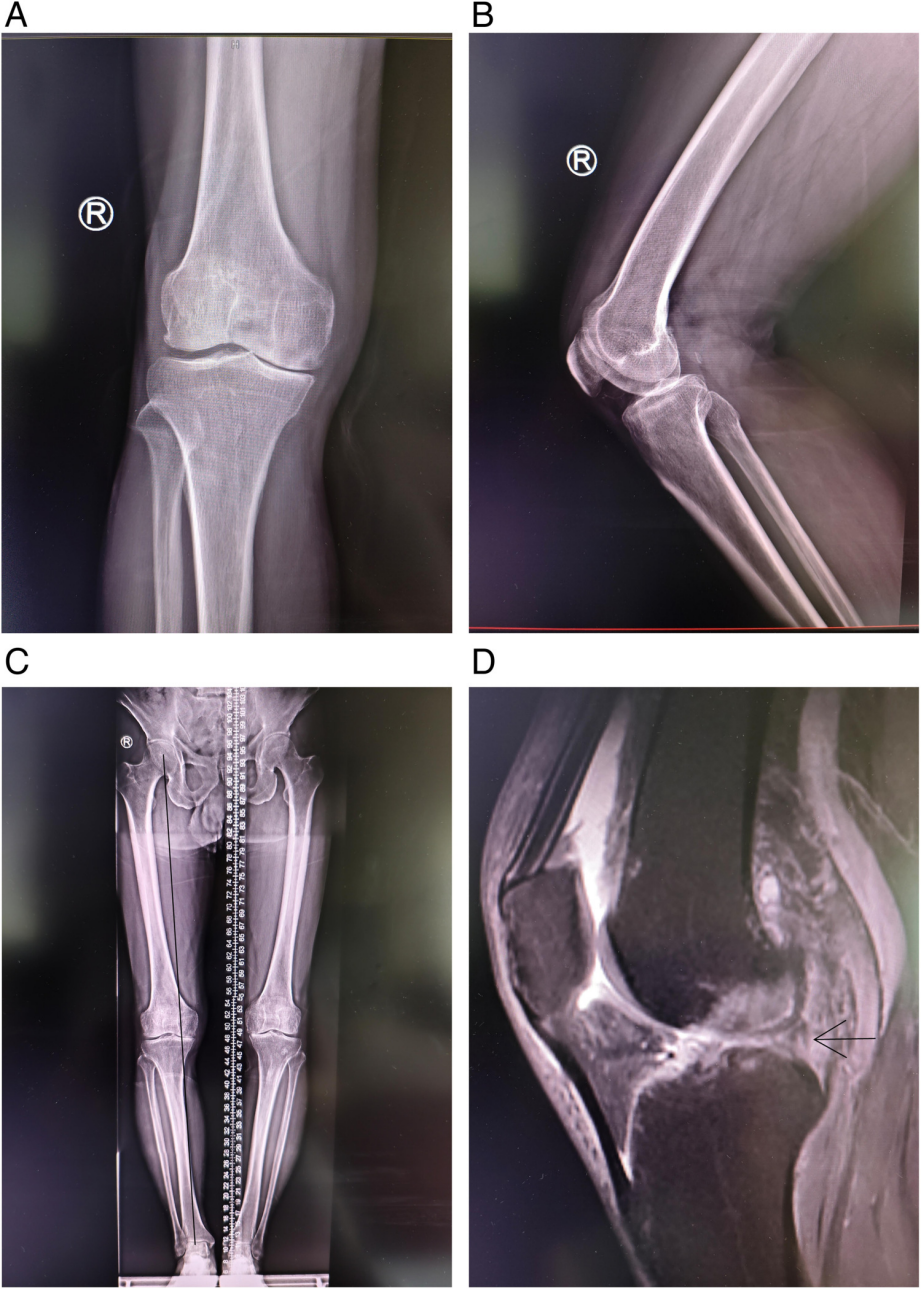
Preoperative X-ray and MRI scans of the right knee joint. **(A,B)** Anteroposterior and lateral X-ray; **(C)** the right lower limb was markedly varus; **(D)** MRI revealed a tear in the posterior root of the lateral meniscus (marked by red arrow). MRI, magnetic resonance imaging.

Based on the patient's symptoms and physical examination, we performed bilateral knee X-rays and magnetic resonance imaging (MRI) of the right knee. Radiographs showed a shorter right lower limb than the left lower limb; osteophytes in the bilateral femoral condyles, intercondylar ridge, tibial plateau, and patellar rim; uneven narrowing of the bilateral knee joint spaces; normal hip and ankle joints; and obvious genu varum of the right lower limb (Figure 1A–C**)**. MRI showed degeneration of the right knee joint, osteochondral damage and bone marrow edema of the medial femoral condyle and medial tibial plateau, effusion in the joint cavity and suprapatellar bursa, and a tear in the posterior horn of the lateral meniscus (Figure 1D). A dedicated axial patellar radiograph was not obtained, as the preoperative MRI already provided comprehensive assessment of the patellofemoral joint, revealing a normal patellar tracking and well-preserved cartilage on both the trochlear and patellar surfaces (Supplementary Figures S1A,B).

The patient's final diagnosis was “osteoarthritis of both knees with meniscus injury of the right knee.”

### 2.3 Therapeutic intervention

**Figure 2:**
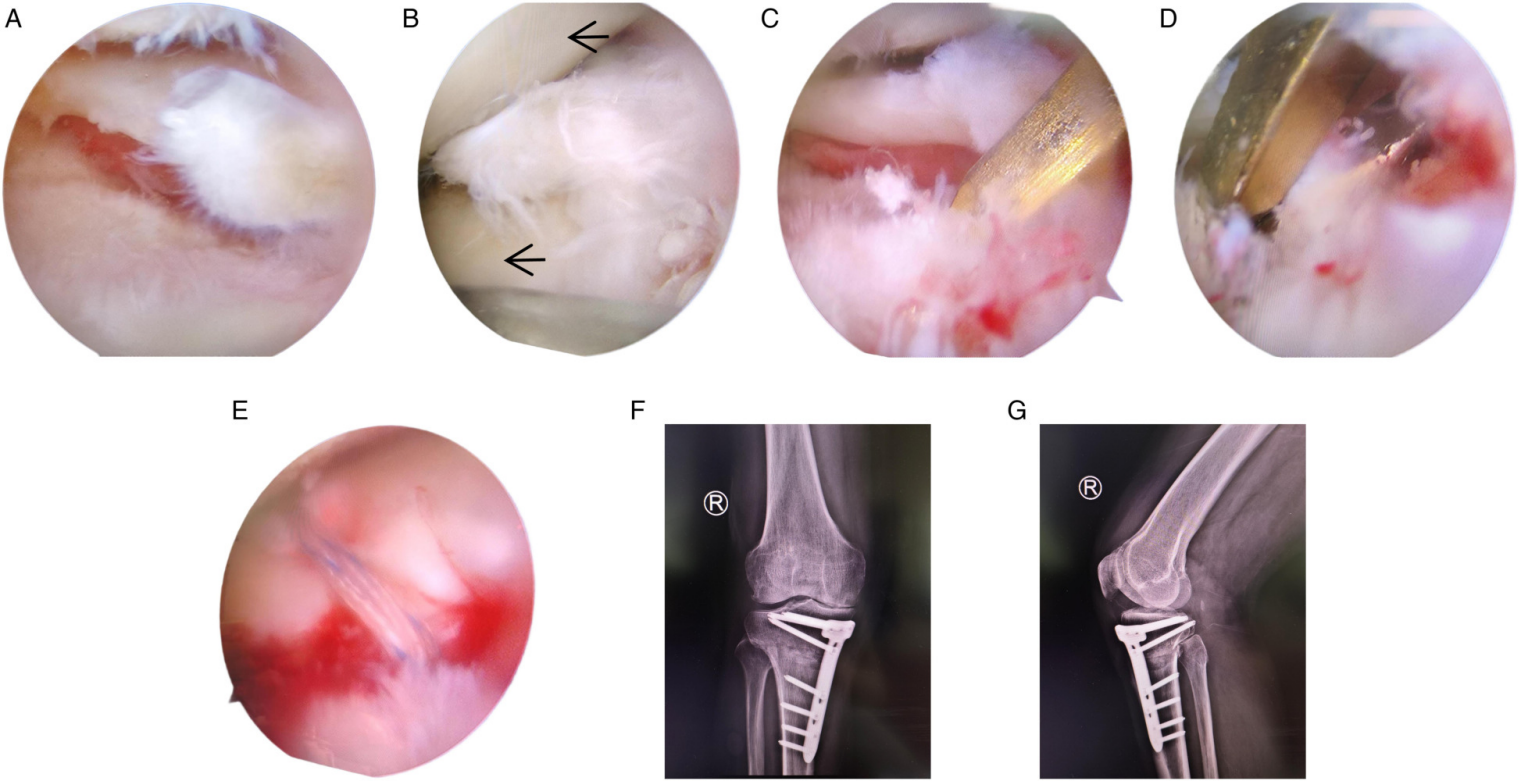
Intraoperative arthroscopic findings and immediate postoperative radiographs. **(A)** The meniscus was torn at the attachment point of the posterior root; **(B)** cartilage in the lateral compartment of the knee is well preserved (marked by red arrows); **(C,D)** two bone tunnels were made at the meniscus attachment using a locator; **(E)** the meniscus sewing thread pulls out of the bone tunnel; **(F,G)** postoperative radiographs, showing that the osteotomy was fixed with a TomoFix plate.

Surgical treatment was performed on 31 January 2023, which included arthroscopic joint cleaning, lateral meniscus attachment reconstruction, and HTO. During surgery, we found that the lateral meniscus was torn from the posterior root, while the meniscus had no hoop stress function and had completely lost its physiological effects (Figure 2A). However, the patient's entire lateral compartment cartilage was well preserved (Figure 2B). At the same time, we also carefully examined the patellofemoral joint and found that the cartilage on the femoral trochlea and patella surfaces was largely normal, showing no signs of chondromalacia or defects. Hence, we created two 3.5-mm bone tunnels with an anterior cruciate ligament (ACL) reconstruction locator at the lateral meniscus attachment point and used a suture hook to suture the posterior root of the meniscus with high-strength sutures. Then, the free ends of the suture were pulled out of the bone tunnels, and were temporarily left unknotted and fixed, so as not to interfere with subsequent operations (Figure 2C–E). A biplane osteotomy of the proximal tibia was performed on the medial side of the tibial tuberosity via an oblique incision and expanded to the appropriate height according to preoperative measurements. Once the correction was seen to reach the predetermined angle under C-arm fluoroscopy (11), fixation was performed using a TomoFix plate (Figure 2F,G). Finally, the meniscus sutures reserved in the bone tunnels were knotted on the bone surface. After surgery, routine treatments, such as anti-infection, swelling reduction, anticoagulation, and rehabilitation, were performed.

### 2.4 Follow-up and outcomes

**Figure 3:**
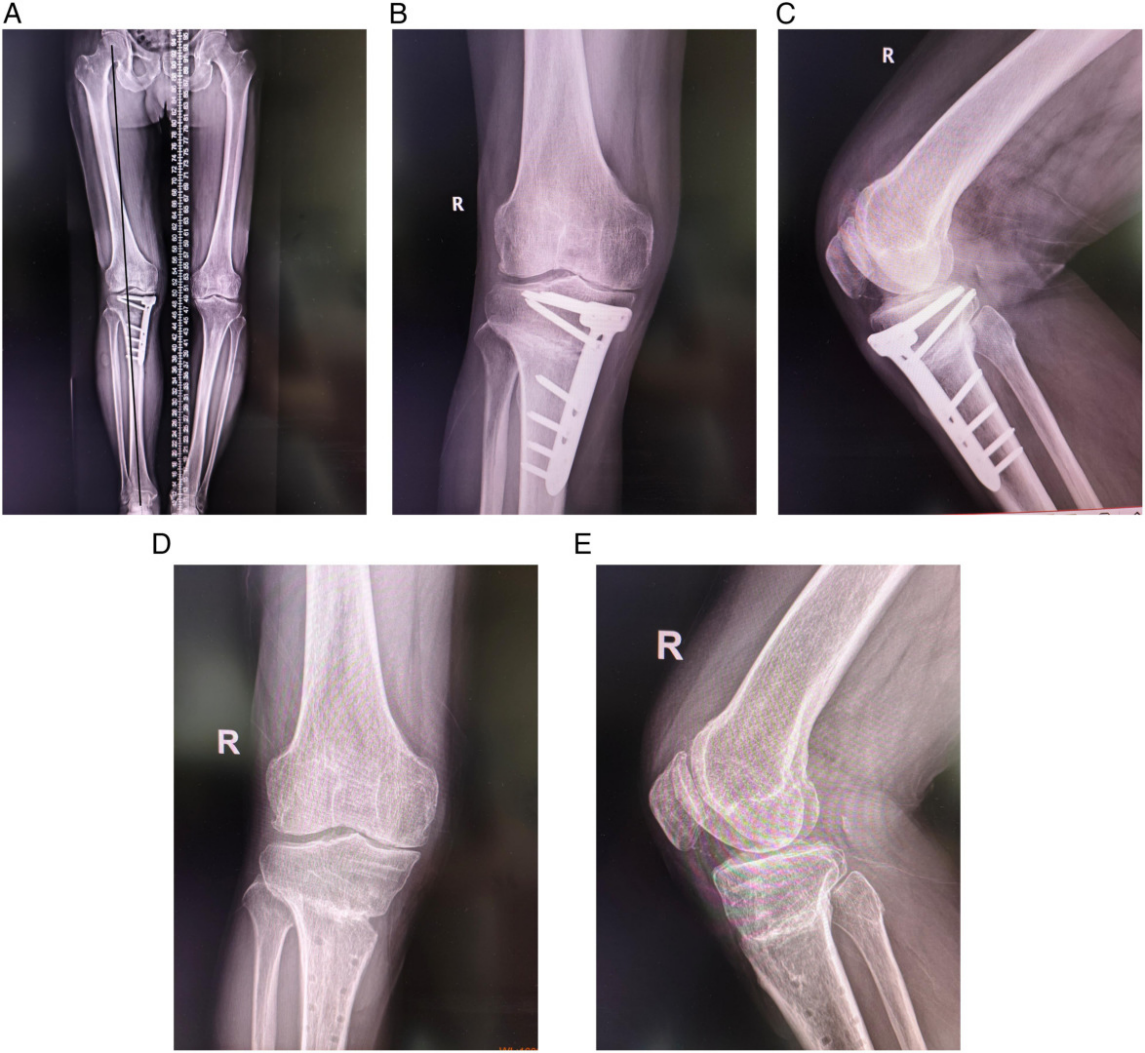
The imaging data were reviewed at each stage after operation. **(A)** Full-length radiographs of both lower limbs 5 days after surgery showed good orthopedic results; **(B,C)** 6 months postoperatively, X-ray films showed that the osteotomy site healed well; **(D,E)** the plate for internal fixation was removed 1 year after surgery.

**Figure 4:**
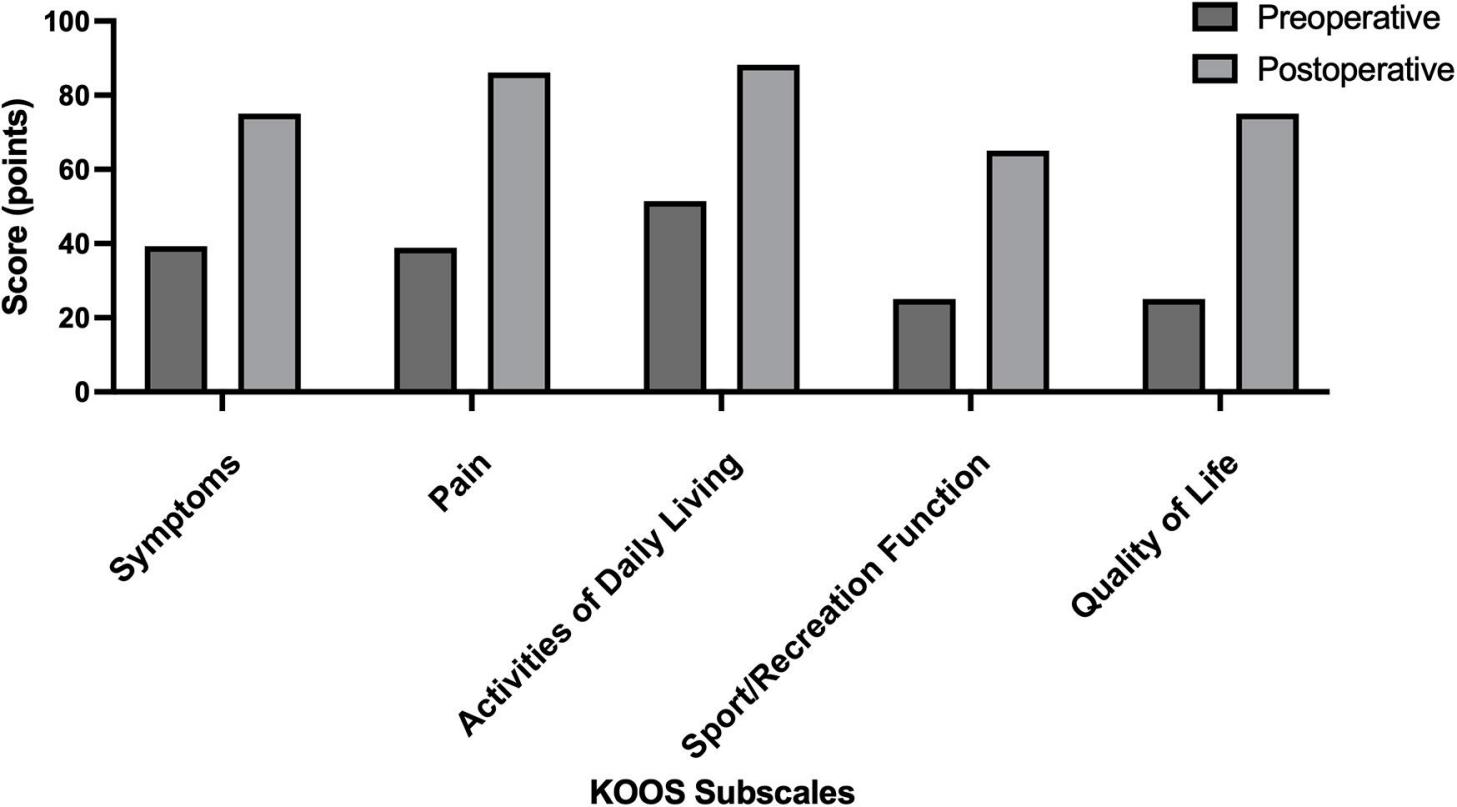
Comparison of KOOS scores before surgery and 1 year after surgery. All five subscales of the KOOS scores showed significant improvement.

On the first postoperative day, the patient's respiratory and circulatory systems were stable, and he exhibited a VAS score of 4. Cefuroxime sodium was administered prophylactically until 24 h postoperatively. On the second postoperative day, electrocardiogram monitoring, urinary catheterization, and oxygenation were discontinued. The patient walked with crutches and with the right knee protected by an offloading brace. On the third postoperative day, functional exercises for the right lower limb were initiated under the supervision of a rehabilitation physician. On the fifth postoperative day, the X-ray was reviewed, showing good orthopedic correction effect and stable internal fixation (Figure 3A). Preoperatively, we planned to correct the lower limb vertical line to the classic Fujisawa point. The patient's preoperative vertical line passed through the medial 25% of the tibial plateau, while the corrected vertical line passed through the lateral 61% of the tibial plateau (Supplementary Figure S2A,B). The patient presented with 8° of genu varum preoperatively and 2° of genu valgum postoperatively (Supplementary Figure S2C,D). Repeat blood count and inflammatory indexes were gradually normalizing and the patient was discharged. At the 6-month postoperative follow-up, the osteotomy site had healed well and the patient could walk normally without aids (Figure 3B,C). The internal fixation plate was removed at the patient's request 1 year postoperatively (Figures 3D,E). When he was hospitalized for steel plate removal, we reassessed his KOOS score (S = 75, *p* = 86.11, ADL = 88.24, Sport = 65, QOL = 75). Compared to his preoperative status, the KOOS score showed significant improvement (Table 2 and Figure 4).

**Table 2:** Preoperative and postoperative KOOS scores.

Tests	Preoperative	Postoperative
Symptoms	39.29	75
Pain	38.89	86.11
Activities of daily living	51.47	88.24
Sport/recreation function	25	65
Quality of life	25	75

## 3 Discussion

Currently, HTO is the preferred and optimal treatment for medial unicompartmental osteoarthritis of the knee and is an important component of “knee preservation” strategy. HTO has significant advantages in correcting knee deformity, alleviating pain, and improving mobility, offering the chance to avoid or delay total knee replacement (12–15). Owing to these benefits, HTO is widely accepted by patients and physicians (16, 17). However, a strict grasp of its indications and contraindications is required for a favorable prognosis (18). Conventionally, patients with lateral meniscus injuries are not considered suitable for HTO, especially if the meniscus is deficient. This is because the principle behind HTO involves shifting the weight from the original medial compartment to the lateral compartment by correcting the weightbearing line, which requires the cartilage and meniscus of the lateral compartment to remain intact. Although concomitant meniscal injuries are common in patients suitable for HTO, the vast majority of injuries occur in the medial meniscus and are due to overstressing of the medial compartment caused by the varus knee (19, 20). At present, the prevailing technique involves arthroscopically examining the lateral compartment of the knee, and cleaning and repairing the medial compartment (including the meniscus) before performing HTO (21, 22). Here, we present the first documented case of a genu varum patient with a traumatic tear in the posterior root of the lateral meniscus, who underwent lateral meniscus attachment reconstruction to restore the integrity of the lateral compartment structures, followed by conventional HTO.

The posterior root of the meniscus plays an important role in maintaining meniscus annular tension and joint stability. When the knee is flexed 90°, the posterior root transmits most of the body load, while its axial loads cannot be converted into annular stresses and transmitted to the tibia after injury (23, 24). Therefore, posterior root tears of the meniscus should be aggressively repaired (25, 26). Current research on posterior meniscus root injuries primarily focuses on the medial meniscus, which is more commonly affected and accounts for approximately 20% of all meniscal injuries due to its higher incidence (27). This may be attributed to the inward alignment of the lower limb force vector in middle-aged and elderly individuals. Although the incidence of posterior lateral meniscus root injury is lower compared to medial meniscus root injuries, the structural integrity of the lateral meniscus remains crucial for knee function. The lateral meniscus has a greater range of motion, and its stability is primarily supported by the anterior and posterior root attachments. Damage to the root attachment points significantly increases the stress across the tibiofemoral joint (28). Studies have shown that in patients with anterior cruciate ligament (ACL) tears, injury to the posterior root of the lateral meniscus leads to a positive pivot shift test, which reflects the knee joint's antero-external rotational stability. Patients with antero-external rotational instability may experience accelerated joint degeneration, chronic pain, and other debilitating symptoms, which require prompt intervention (29). Damage to the posterior root of the lateral meniscus results in the loss of the protective ring effect in the lateral compartment, altering knee joint kinematics. Under pressure, the lateral meniscus may become displaced or even dislocated, further compromising normal function. In conclusion, injury to the posterior root of the lateral meniscus can have significant repercussions, including destabilization of the knee joint, increased risk of osteoarthritis, and the development of chronic pain, swelling, and functional impairment. Early diagnosis and treatment are essential to prevent these outcomes, with active surgical repair often being the recommended approach.

Historically, meniscoplasty was the primary treatment for posterior meniscus root injury, but it was associated with numerous long-term complications and has since been largely replaced. Currently, the main repair techniques include suture anchoring, tibial tunnel suturing, and edge-to-edge suturing, each with specific indications based on the nature of the injury. The edge-to-edge suture method typically involves using a total internal suturing device to secure the torn root ends. This technique requires sufficient, high-quality meniscal tissue on both sides of the tear, making it relatively straightforward to perform. It also has a short learning curve, making it particularly suitable for less experienced surgeons or those new to this type of procedure (30). The use of suture anchor repair is relatively limited due to its restricted operating space, the technical difficulty of anchor placement, and the potential risk of neurovascular injury associated with the approach. As a result, this technique is primarily utilized for meniscus root repair in cases where multiple ligaments are injured (31). The tibial tunnel suture technique, first introduced by Raustol in 2006 (32), involves preparing a bone tunnel at the injury site using the ACL reconstruction locator. The meniscus root is then secured to the bone through the tunnel using sutures (33). This method provides strong fixation and is suitable for a variety of posterior meniscus root injuries. However, it requires precise suture placement and an adequate number of sutures; otherwise, improper suturing can lead to suture failure and secondary tissue damage. In this case, we used a third technique for repairing meniscal injuries—transtibial tunnel attachment reconstruction. Since this case involved a radial tear at the posterior root of the meniscus, the stump did not retain enough tissue for suturing, and the lateral meniscus would need to bear greater stress after HTO. Hence, a stronger, more reliable method of repair was needed. Compared with the commonly used all-inside suture (Fast-Fix suture system), the pull-out technique has a higher repair strength and can anchor the meniscus vertically downward to the bone surface, so that the repaired meniscus is less likely to protrude outward. This basically restores its original anatomical position and biomechanics, with satisfactory clinical outcomes (34–36).

Although HTO provides us with an ideal “knee preservation” option, as mentioned earlier, rigorous adherence to the indications is needed to achieve a good outcome. The majority of patients encountered in the clinic are reluctant to undergo total knee replacement, which prompted us to consider how to scientifically expand the indications for HTO and reduce the number of people undergoing joint replacement. This case is an attempt guided by this concept. The patient was initially considered suitable for HTO, but the trauma that occurred 1 month before admission caused a tear at the posterior root of the lateral meniscus. Such patients are traditionally not considered to meet the conditions for HTO, but the patient was very resolute in refusing joint replacement. Therefore, under the premise of achieving a strong repair of the meniscus, we proceeded with HTO. The patient demonstrated excellent efficacy at the 1-year postoperative follow-up. Hence, our attempt has expanded the indications for HTO and avoided the need for joint replacement.

Naturally, not all patients are suitable for expanded indications through targeted management; we believe that this approach is limited to cases where the cartilage of the lateral compartment is intact. Some structural injuries due to acute trauma, such as cruciate ligament rupture, acute osteochondral injuries, and acute meniscal injuries, allow us to create conditions suitable for HTO surgery through cruciate ligament reconstruction, osteochondral grafting, and meniscal repair (37, 38). The above structural repair surgery can be performed simultaneously with HTO in one stage or separately in two stages, and the timing of the surgery can be chosen according to the patient's condition and wishes. However, most patients may choose to undergo the procedures simultaneously in one stage for economic reasons. The postoperative rehabilitation method of this structural repair surgery combined with HTO is different from that of conventional HTO. For example, in patients who undergo HTO alone, early mobilization, along with strengthening flexion and extension exercises, are generally required. Patients are also required to use crutches for 1 month after surgery and may resume full weightbearing walking after 1 month. However, in this case involving meniscal attachment reconstruction combined with HTO, we asked the patient to limit his knee flexion to 90° for 4 weeks, wear a brace to allow full range of motion of the joint for 8 weeks, and only resume full weightbearing walking after 8 weeks, with no deep squatting for half a year. This is clearly a more conservative rehabilitation program than that for HTO alone and aims to ensure effective healing of the meniscus.

One limitation of this study is the use of the KOOS alone as a patient-reported outcome measure. Although the KOOS comprehensively captures pain, symptoms, and quality of life relevant to this condition, the concurrent use of a joint-specific score like the Lysholm score could have provided additional validation and a more focused assessment of functional stability. This is an area we need to improve upon in future similar studies. Another limitation is that this is merely a single case report without a control group to validate the advantages of our surgical approach. It only compares the patient's preoperative and postoperative outcomes. We will continue to collect similar cases and proceed to conduct a comparative study of different treatment protocols in the next phase.

## 4 Conclusion

This is a rare and unique case of lateral meniscus attachment reconstruction combined with HTO, in which we innovatively performed a strong repair on the lateral meniscus first to expand the indications for HTO. Subsequent longer-term follow-up of this case and more similar cases are needed to support this conclusion.

## Data Availability

The original contributions presented in the study are included in the article/Supplementary Material, further inquiries can be directed to the corresponding authors.

## References

[1] Coventry MB. Osteotomy of the upper portion of the tibia for degenerative arthritis of the knee: a preliminary report. J Bone Joint Surg Am. (1965) 47:984–90. 10.2106/00004623-196547050-00008 14318636

[2] Maquet P. Valgus osteotomy for osteoarthritis of the knee. Clin Orthop Relat Res. (1976) 120:143–8. 10.1097/00003086-197610000-00022 975649

[3] Aydogdu S Cullu E Araç N Varolgüneş N Sur H. Prolonged peroneal nerve dysfunction after high tibial osteotomy: pre- and postoperative electrophysiological study. Knee Surg Sport Tr A. (2000) 8:305–8. 10.1007/s001670000138 11061301

[4] Perren SM. Evolution of the internal fixation of long bone fractures: the scientific basis of biological internal fixation: choosing a new balance between stability and biology. J Bone Joint Surg Br. (2002) 84:1093–110. 10.1302/0301-620x.84b8.13752 12463652

[5] Staubli A Simoni C Babst R Lobenhoffer P. Tomofix: a new LCP-concept for open wedge osteotomy of the medial proximal tibia—early results in 92 cases. Injury. (2003) 34(supplement 2):55–62. 10.1016/j.injury.2003.09.025 14580986

[6] Agneskirchner J Freiling D Hurschler C Lobenhoffer P. Primary stability of four different implants for opening wedge high tibial osteotomy. Knee Surg Sport Tr A. (2006) 14:291–300. 10.1007/s00167-005-0690-1 16284740

[7] Coventry MB. Upper tibial osteotomy for gonarthrosis: the evolution of the operation in the last 18 years and long term results. Orthop Clin N Am. (1979) 10:191–210. 10.1016/S0030-5898(20)30585-X 450397

[8] Coventry MB. Upper tibial osteotomy. Clin Orthop Relat R. (1984) 182:46–52. 10.1097/00003086-198401000-00008 6692627

[9] Lobenhoffer P De Simoni C Staubli A. Open-wedge high-tibial osteotomy with rigid plate fixation. J Knee Surg. (2002) 1:93–105. 10.1097/00132588-200212000-00004

[10] Outerbridge RE. The etiology of chondromalacia patellae. J Bone Joint Surg Br. (1961) 43, 752–7 10.1302/0301-620X.43B4.752 14038135

[11] Fujisawa Y Masuhara K Shiomi S. The effect of high tibial osteotomy on osteoarthritis of the knee. An arthroscopic study of 54 knee joints. Orthop Clin N Am. (1979) 10:585–608. 10.1016/S0030-5898(20)30753-7 460834

[12] Windsor RE Insall JN Vince KG. Technical considerations of total knee arthroplasty after proximal tibial osteotomy. J Bone Joint Surg Am. (1988) 70:547–55. 10.2106/00004623-198870040-00011 3356722

[13] Odenbring S Tjörnstrand B Egund N Hagstedt B Hovelius L Lindstrand A Function after tibial osteotomy for medial gonarthrosis below aged 50 years. Acta Orthop Scand. (1989) 60:527–31. 10.3109/17453678909150116 2603651

[14] Nagel A Insall JN Scuderi GR. Proximal tibial osteotomy. A subjective outcome study. J Bone Joint Surg Am. (1996) 78:1353–8. 10.2106/00004623-199609000-00009 8816650

[15] Han JH Yang JH Bhandare NN Suh DW Lee JS Chang YS Total knee arthroplasty after failed high tibial osteotomy: a systematic review of open versus closed wedge osteotomy. Knee Surg Sport Tr A. (2016) 24:2567–77. 10.1007/s00167-015-3807-1 26423055

[16] Ritter MA Fechtman RA. Proximal tibial osteotomy. A survivorship analysis. J Arthroplasty. (1988) 3:309–11. 10.1016/s0883-5403(88)80030-5 3241167

[17] Berman AT Bosacco SJ Kirshner S Avolio A Jr. Factors influencing long-term results in high tibial osteotomy. Clin Orthop Relat R. (1991) 272:192–8. 10.1097/00003086-199111000-00028 1934732

[18] Lu J Tang S Wang Y Li Y Liu C Niu Y Clinical outcomes of closing- and opening-wedge high tibial osteotomy for treatment of anteromedial unicompartmental knee osteoarthritis. J Knee Surg. (2019) 32:758–63. 10.1055/s-0038-1668124 30103219

[19] Sharma L Chmiel JS Almagor O Felson D Guermazi A Roemer F The role of varus and valgus alignment in the initial development of knee cartilage damage by MRI: the MOST study. Ann Rheum Dis. (2013) 72:235–40. 10.1136/annrheumdis-2011-201070 22550314 PMC3845483

[20] Sharma L Chang AH Jackson RD Nevitt M Moisio KC Hochberg M Varus thrust and incident and progressive knee osteoarthritis. Arthritis Rheumatol. (2017) 69:2136–43. 10.1002/art.40224 28772066 PMC5659924

[21] Yoo MJ Shin YE. Open wedge high tibial osteotomy and combined arthroscopic surgery in severe medial osteoarthritis and Varus malalignment: minimum 5-year results. Knee Surg Relat Res. (2016) 28:270–6. 10.5792/ksrr.15.075 27894173 PMC5134789

[22] Carreau JH Sitton SE Bollier M. Medial meniscus root tear in the middle aged patient: a case based review. Iowa Orthop J. (2017) 37:123. 28852346 PMC5508273

[23] Allaire R Muriuki M Gilbertson L Harner CD. Biomechanical consequences of a tear of the posterior root of the medial meniscus. Similar to total meniscectomy. J Bone Joint Surg Am. (2008) 90:1922–31. 10.2106/JBJS.G.00748 18762653

[24] Fox AJ Bedi A Rodeo SA. The basic science of human knee menisci: structure, composition, and function. Sports Health. (2012) 4:340–51. 10.1177/1941738111429419 23016106 PMC3435920

[25] Laprade CM Jansson KS Dornan G Smith SD Wijdicks CA Laprade RF. Altered tibiofemoral contact mechanics due to lateral meniscus posterior horn root avulsions and radial tears can be restored with *in situ* pull-out suture repairs. J Bone Joint Surg Am. (2014) 96:471–9. 10.2106/JBJS.L.01252 24647503

[26] Ohori T Mae T Shino K Fujie H Hirose T Tachibana Y Different effects of the lateral meniscus complete radial tear on the load distribution and transmission functions depending on the tear site. Knee Surg Sport Tr A. (2021) 29:342–51. 10.1007/s00167-020-05915-8 32152692

[27] Bin S-I Kim J-M Shin S-J. Radial tears of the posterior horn of the medial meniscus. Arthroscopy. (2004) 20:373–8. 10.1016/j.arthro.2004.01.004 15067276

[28] Schillhammer CK Werner FW Scuderi MG Cannizzaro JP. Repair of lateral meniscus posterior horn detachment lesions: a biomechanical evaluation. Am J Sports Med. (2012) 40:2604–9. 10.1177/0363546512458574 22972853

[29] Jonsson H Riklund-Åhlström K Lind J. Positive pivot shift after ACL reconstruction predicts later osteoarthrosis 63 patients followed 5–9 years after surgery. Acta Orthop Scand. (2004) 75:594–9. 10.1080/00016470410001484 15513493

[30] Zhuo H Chen Q Zhu F Li J. Arthroscopic side-to-side repair for complete radial posterior lateral meniscus root tears. BMC Musculoskel Dis. (2020) 21:1–6. 10.1186/s12891-020-3156-1 PMC7049216 32111224

[31] Lee Y-B Yang C-J Li CZ Zhuan Z Kwon S-C Noh K-C Medial meniscal root repair using curved guide and soft suture anchor. Clin Orthop Surg. (2018) 10:111–5. 10.4055/cios.2018.10.1.111 29564055 PMC5851846

[32] Raustol OA Poelstra KA Chhabra A Diduch DR. The meniscal ossicle revisited: etiology and an arthroscopic technique for treatment. Arthroscopy. (2006) 22:687.e1. 10.1016/j.arthro.2005.12.022 16762719

[33] Masuda S Furumatsu T Okazaki Y Kamatsuki Y Okazaki Y Kodama Y Transtibial pullout repair reduces posterior extrusion of the medial meniscus. Acta Med Okayama. (2019) 73:495–501. 10.18926/AMO/57713 31871331

[34] Forkel P Petersen W. Posterior root tear fixation of the lateral meniscus combined with arthroscopic ACL double-bundle reconstruction: technical note of a transosseous fixation using the tibial PL tunnel. Arch Orthop Traum Su. (2012) 132:387–91. 10.1007/s00402-011-1429-8 22080932

[35] Jiang EX Everhart JS Abouljoud M Kirven JC Magnussen RA Kaeding CC Biomechanical properties of posterior meniscal root repairs: a systematic review. Arthroscopy. (2019) 35:2189–206. 10.1016/j.arthro.2019.01.018 30979628

[36] Okazaki Y Furumatsu T Kamatsuki Y Okazaki Y Masuda S Hiranaka T Transtibial pullout repair of the lateral meniscus posterior root tear combined with anterior cruciate ligament reconstruction reduces lateral meniscus extrusion: a retrospective study. Orthop Traumatol-Sur. (2020) 106:469–73. 10.1016/j.otsr.2019.10.022 32278734

[37] Kwon SK Moon HK Choi CJ Park SH Lee JJ Kim YC Accelerated degeneration of the discoid lateral meniscus after medial opening wedge high tibial osteotomy. Knee Surg Sport Tr A. (2015) 23:97–103. 10.1007/s00167-012-2289-7 23188498

[38] Prakash J Song EK Lim HA Shin YJ Jin C Seon JK. High tibial osteotomy accelerates lateral compartment osteoarthritis in discoid meniscus patients. Knee Surg Sport Tr A. (2018) 26:1845–50. 10.1007/s00167-017-4422-0 28160013

