# Supplementary figures and images for: Meniscal attachment reconstruction combined with high tibial osteotomy in a patient with genu varum and posterior root injury of the lateral meniscus: a case report and brief review of the literature
Source: Front Surg. 2025 Dec 29;12:1719884. doi: 10.3389/fsurg.2025.1719884 (PMC12793105; doi:10.3389/fsurg.2025.1719884)

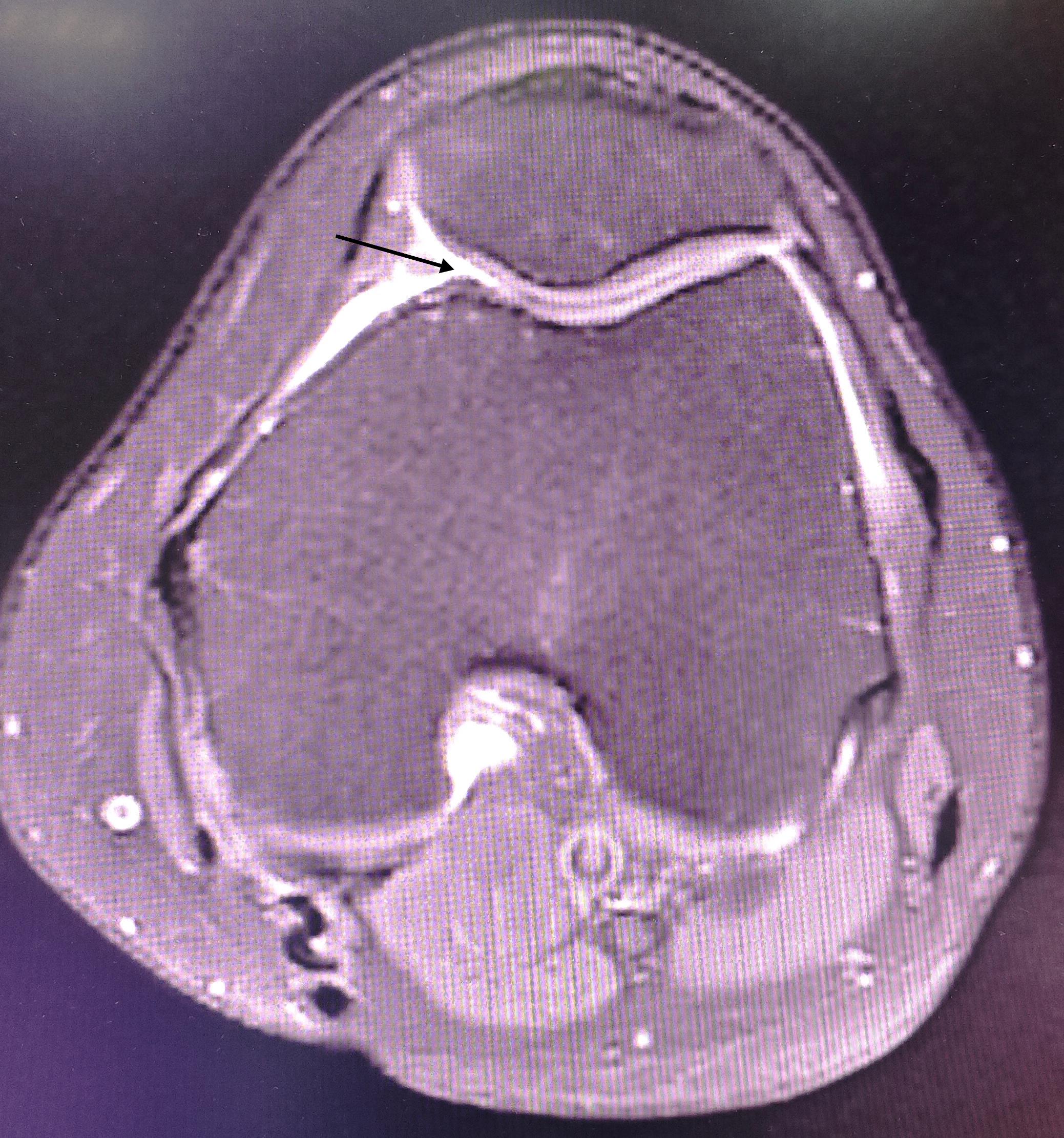

Supplement: Supplementary file 1 [file Supplementaryfile1.zip › Supplementary Figure S1/Supplementary Figure S1(B).jpg]

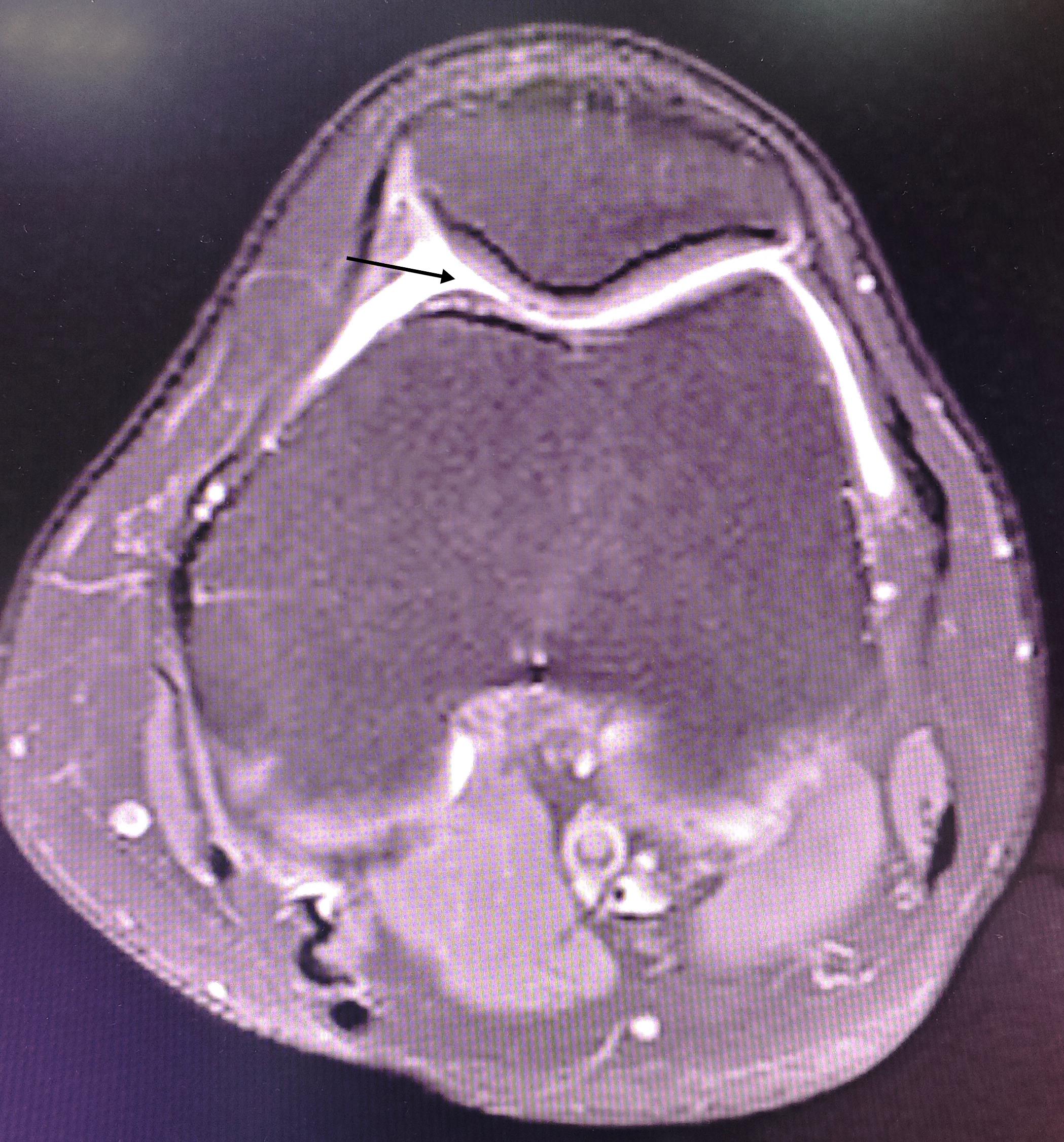

Supplement: Supplementary file 1 [file Supplementaryfile1.zip › Supplementary Figure S1/Supplementary Figure S1(A).jpg]

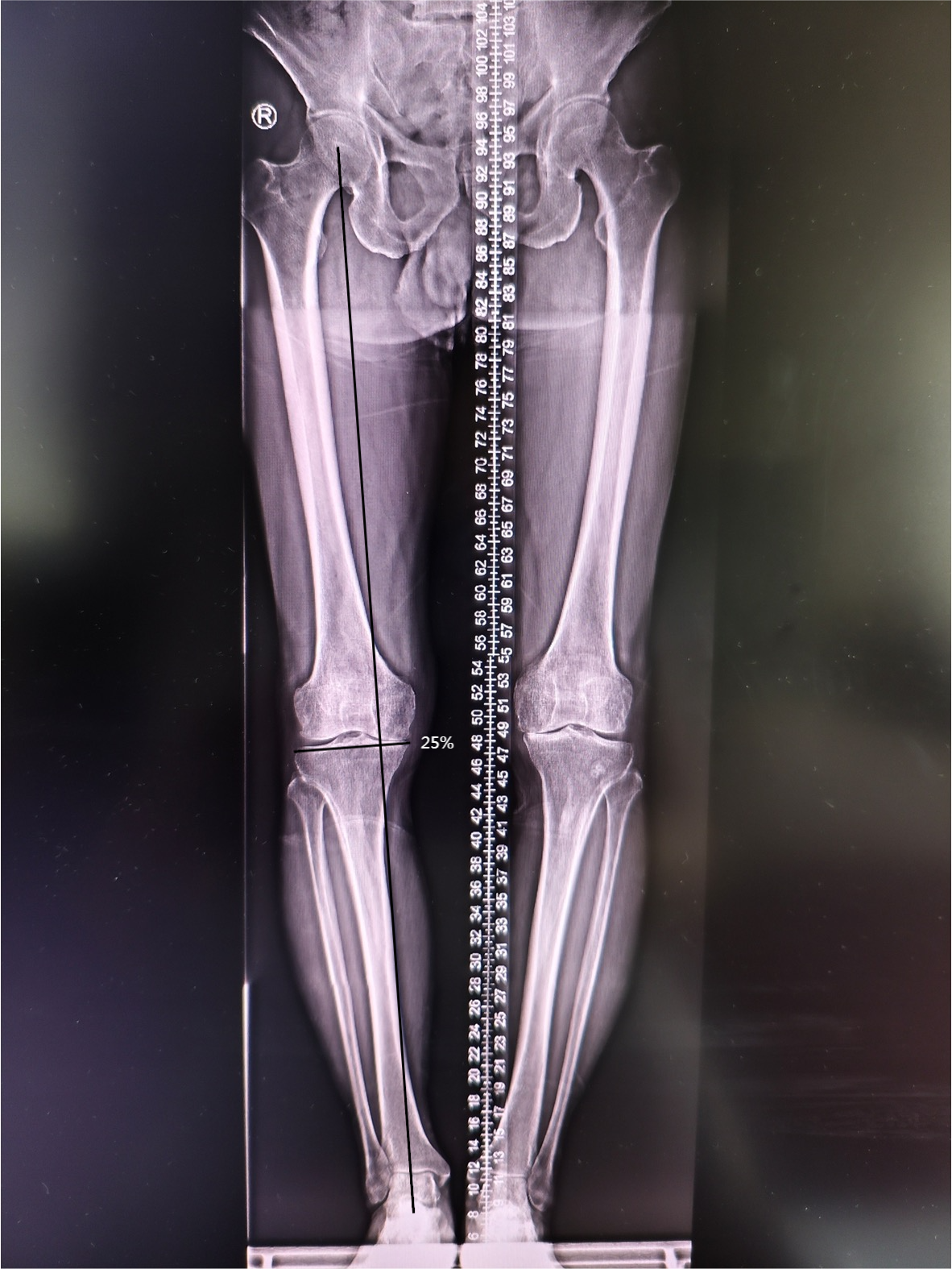

Supplement: Supplementary file 2 [file Supplementaryfile2.zip › Supplementary Figure S2/Supplementary Figure S2(A).jpg]

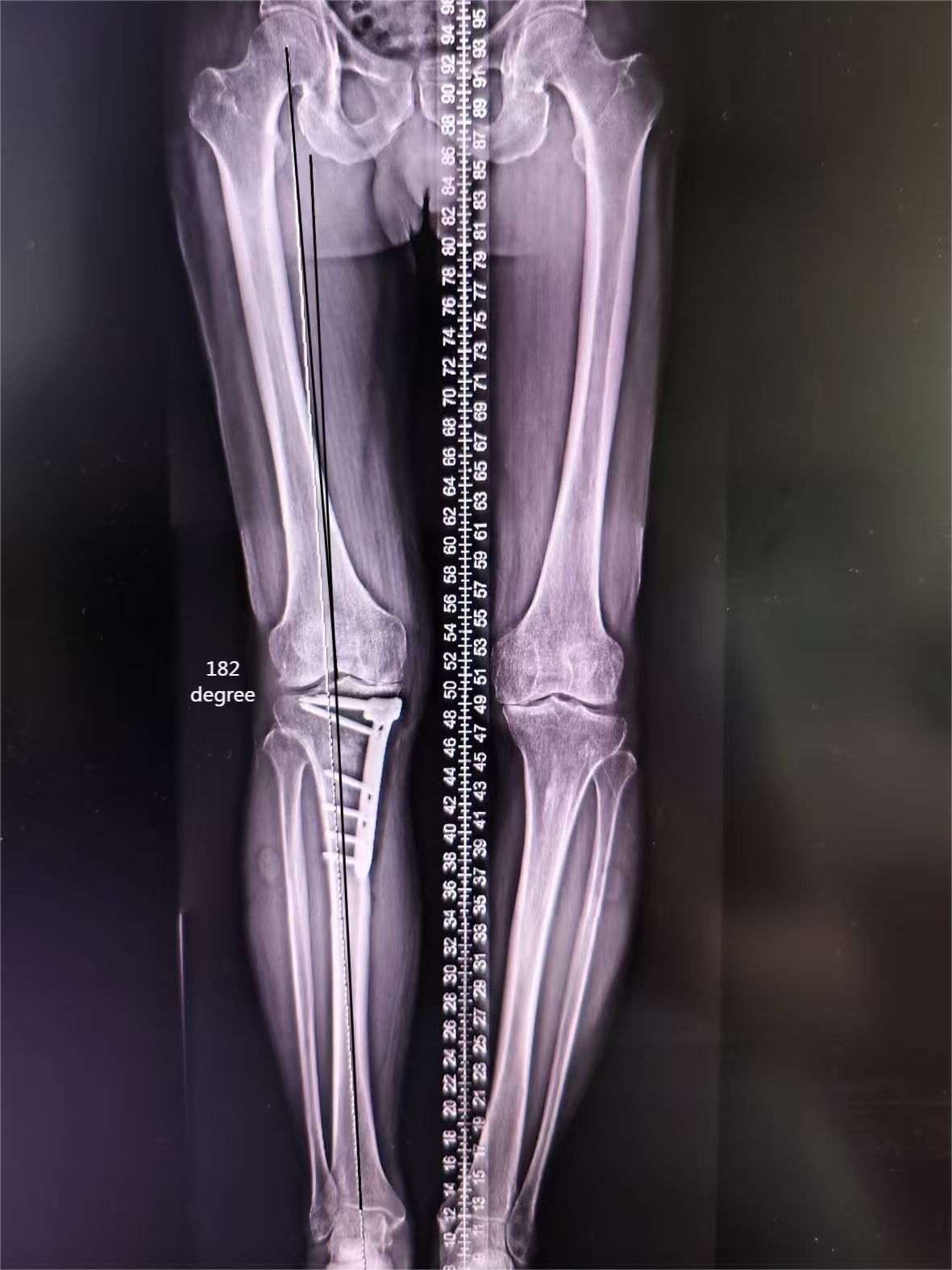

Supplement: Supplementary file 2 [file Supplementaryfile2.zip › Supplementary Figure S2/Supplementary Figure S2(D).jpg]

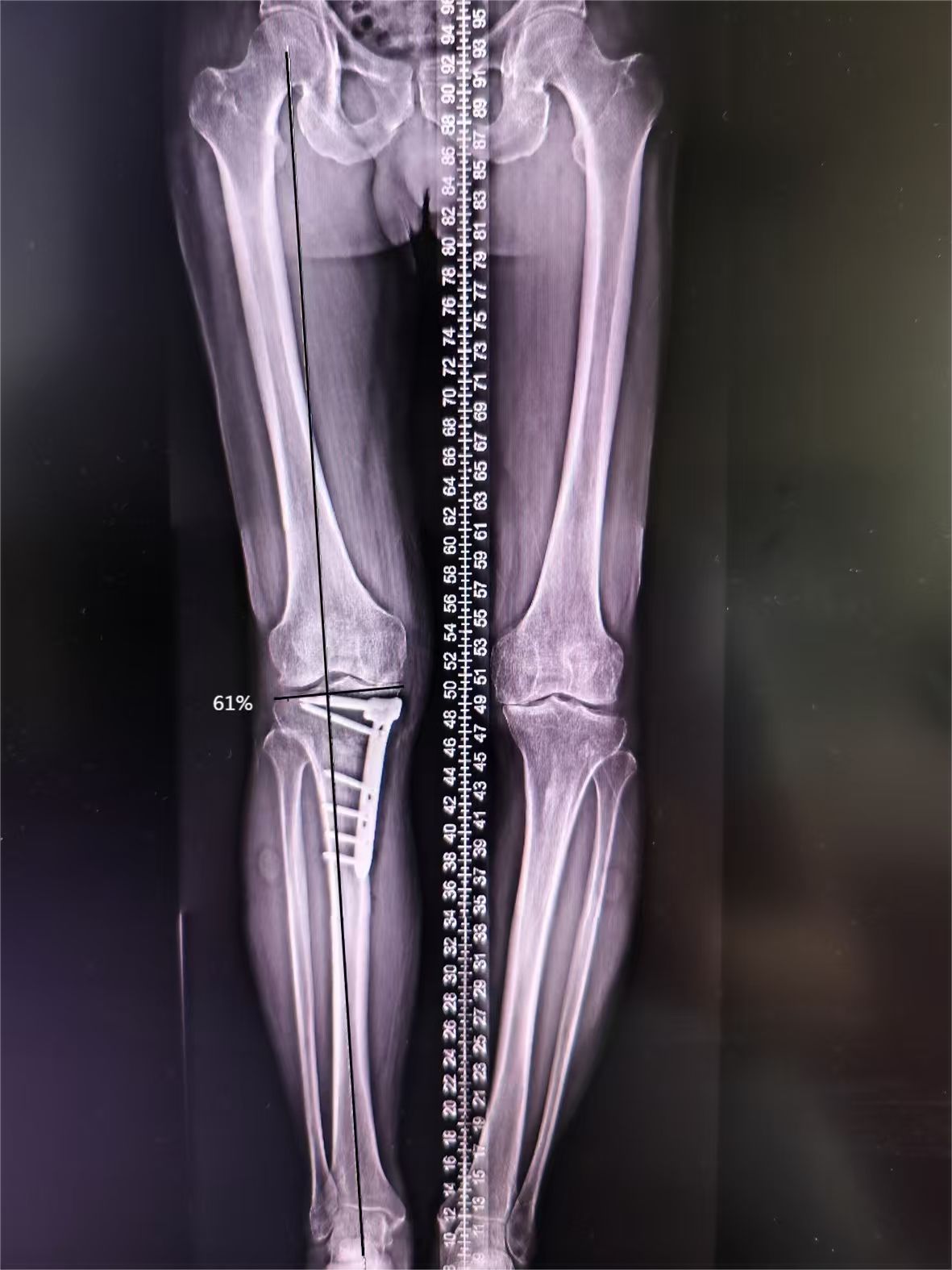

Supplement: Supplementary file 2 [file Supplementaryfile2.zip › Supplementary Figure S2/Supplementary Figure S2(B).jpg]

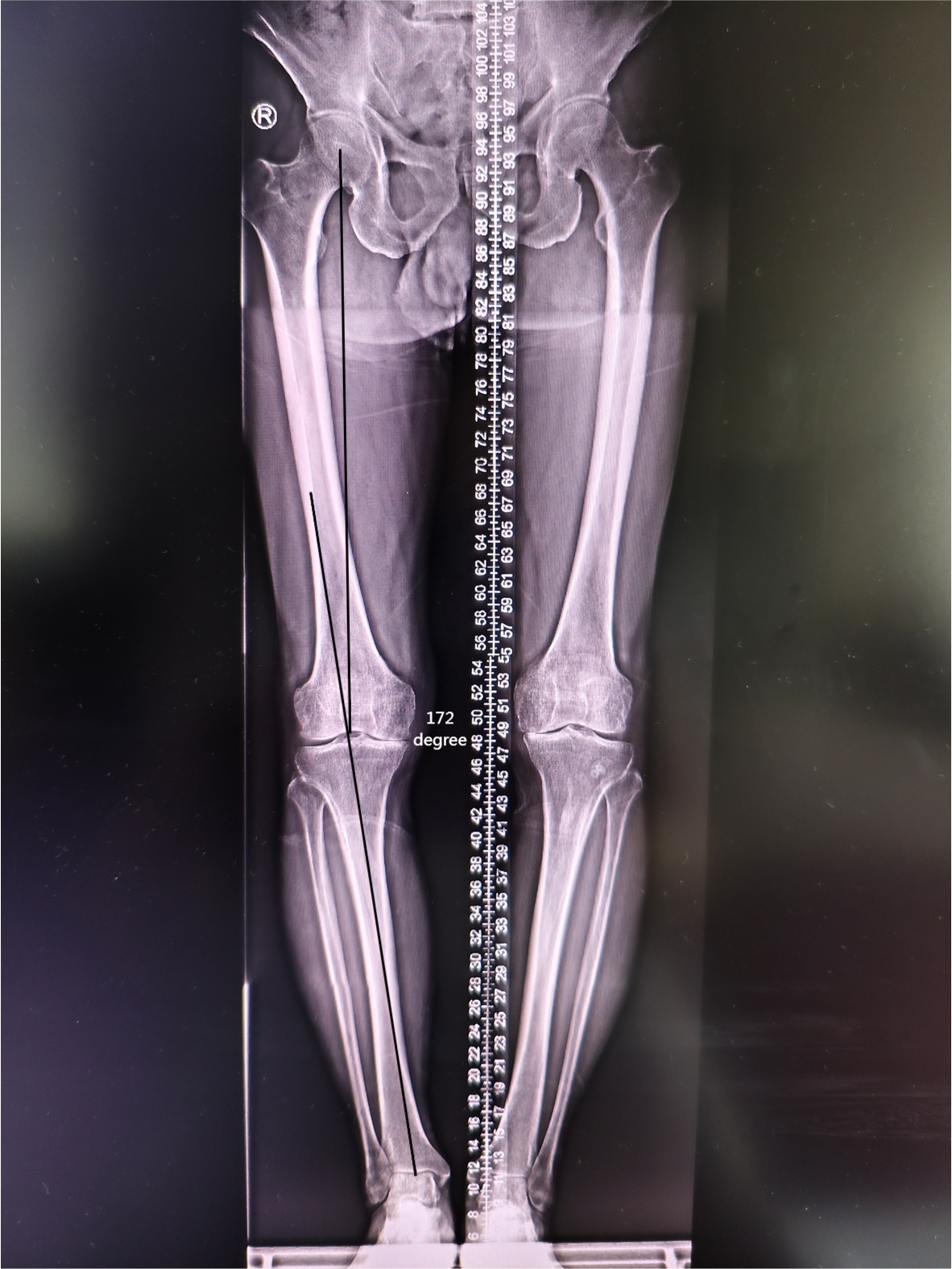

Supplement: Supplementary file 2 [file Supplementaryfile2.zip › Supplementary Figure S2/Supplementary Figure S2(C).jpg]
